# THE STEP-HI STUDY: RECRUITMENT, ADHERENCE, AND RESULTS FOR PRIMARY STUDY OUTCOME

**DOI:** 10.1093/geroni/igae098.0271

**Published:** 2024-12-31

**Authors:** Ellen Binder, Sarah Berry, Jenna Bartley, Robin Marcus, Christine McDonough, Dominic Reeds, Jay Magaziner, Kenneth Schechtman

**Affiliations:** Washington University School of Medicine, St. Louis, Missouri, United States; Hebrew Rehabilitation Center for the Aged, Boston, Massachusetts, United States; UConn Health, Farmington, Connecticut, United States; University of Utah Health, Salt Lake City, Utah, United States; University of Pittsburgh, Pittsburgh, Pennsylvania, United States; Washington University School of Medicine, St. Louis, Missouri, United States; University of Maryland School of Medicine, Baltimore, Maryland, United States; Washington University School of Medicine, St. Louis, Missouri, United States

## Abstract

For STEP-HI women aged ≥65 years with a recent hip fracture were recruited from the community at large and Assisted Living facilities. Women who qualified for the study were assigned to one of three groups for 6 months: exercise plus testosterone (EX+T), exercise plus placebo (EX+P), or Enhanced Usual Care (EUC). EX+T were prescribed a dose of 1% testosterone topical gel intended to maintain serum total testosterone levels between 110-160 ng/dL. Both EX groups performed a supervised exercise program; EUC participants performed home exercise. To identify eligible patients 4695 chart reviews were completed, 207 women consented for screening, 153 were eligible, 129 were randomized to study groups, 122 included in the intention-to-treat (ITT) analyses. Mean age of the sample was 79.3 ± 8.4. Women assigned to the EX+T group achieved a mean serum total testosterone level of 170.1 ± 82.7 ng/dL, compared with 17.8 ± 13.3 ng/dL in the EX+P group (p< 0.001). Adherence to the supervised exercise program (number completed/number of expected sessions) was 82.5% in the EX+T group and 81.1% in the EX+P group (p = 0.68). The pre-specified primary outcome measure was Six Minute Walk Distance (SMWD). In the ITT analysis, SMWD increased over the 6-month intervention period in all three groups, but the changes in EX+T (n=53) vs. EX+P (n=51) vs. EUC (n=18) did not differ significantly by group (p> 0.39 for all comparisons). We conclude that in older women with a recent hip fracture, testosterone replacement therapy does not independently improve SMWD greater than exercise.

